# Antibacterial and wound healing stimulant nanofibrous dressing consisting of soluplus and soy protein isolate loaded with mupirocin

**DOI:** 10.1038/s41598-024-78161-4

**Published:** 2024-11-02

**Authors:** Maryam Jahani, Azadeh Asefnejad, Mastafa H. Al-Musawi, Ahmed A. Mohammed, Basma Talib Al-Sudani, Maha Hameed Al-bahrani, Nada A. Kadhim, Mina Shahriari-Khalaji, Hamideh Valizadeh, Fariborz Sharifianjazi, Morteza Mehrjoo, Ketevan Tavamaishvili, Mohamadreza Tavakoli

**Affiliations:** 1 Department of Biomedical Engineering, Science and Research Branch, Islamic Azad University, Tehran, Iran; 2 Department of Clinical Laboratory Sciences, College of Pharmacy, Mustansiriyah University, Baghdad, Iraq; 3 College of Pharmacy, Branch of Clinical Laboratory Sciences, University of Mustansiriyah, Baghdad, Iraq; 4 Department of Molecular and Medical Biotechnology, College of Biotechnology, Al-Nahrain University, Baghdad, Iraq; 5 Department of Chemistry, College of Science, University of Baghdad, Baghdad, Iraq; 6 Department of Biomedical Engineering, Rowan University, Glassboro, NJ 08028 USA; 7 Department of tissue engineering and regenerative medicine, Faculty of advanced technologies in medicine, Iran University of Medical Sciences, Tehran, Iran; 8 Department of Natural Sciences, School of Science and Technology, University of Georgia, Tbilisi, 0171 Georgia; 9 Department of Biomedical Engineering, Amirkabir University of Technology, Tehran, Iran; 10 School of Medicine, Georgian American University, 10 Merab Aleksidze Str., Tbilisi, 0160 Georgia; 11 Department of Materials Engineering, Isfahan University of Technology, Isfahan, 84156-83111 Iran

**Keywords:** Nanofiber, Wound dressing, Soluplus, Soy protein isolate, Mupirocin, Nanostructures, Biological techniques

## Abstract

Severe cutaneous injuries may not heal spontaneously and may necessitate the use of supplementary therapeutic methods. Electrospun nanofibers possess high porosity and specific surface area, which provide the necessary microenvironment for wound healing. Here in, the nanofibers of Soluplus-soy protein isolate (Sol-SPI) containing mupirocin (Mp) were fabricated via electrospinning for wound treatment. The fabricated nanofibers exhibited water absorption capacities of about 300.83 ± 29.72% and water vapor permeability values of about 821.8 ± 49.12 g/m^2^ day. The Sol/SPI/Mp nanofibers showed an in vitro degradability of 33.73 ± 3.55% after 5 days. The ultimate tensile strength, elastic modulus, and elongation of the Sol/SPI/Mp nanofibers were measured as 3.61 ± 0.29 MPa, 39.15 ± 5.08 MPa, and 59.11 ± 1.94%, respectively. Additionally, 85.90 ± 6.02% of Mp loaded in the nanofibers was released in 5 days in vitro, and by applying the Mp-loaded nanofibers, 93.06 ± 5.40% and 90.40 ± 5.66% of S. aureus and E. coli bacteria were killed, respectively. Human keratinocyte cells (HaCat) demonstrated notable biocompatibility with the prepared nanofibers. Furthermore, compare to other groups, Sol-SPI-Mp nanofibers caused the fastest re-epithelialization and wound healing in a rat model. The findings of this study present a novel nanofiber-based wound dressing that accelerates the healing of severe skin wounds with the risk of infection.

**Supplementary Information:**

The online version contains supplementary material available at 10.1038/s41598-024-78161-4.

## Introduction

Patients with non-healing wounds represent a significant concern in healthcare systems globally. Microbial infections are the primary cause of chronic wounds^1^, including diabetic and venous leg ulcers, as well as burn injuries, all of which pose a significant clinical challenge and result in high healthcare costs. Upon wound formation, opportunistic pathogens can infiltrate the wound environment and form a biofilm, that effectively prevents antibiotics penetration. In this situation, the control of the infection is extremely difficult. Furthermore, the increasing incidence of chronic wound infections caused by antibiotic-resistant pathogens in medical facilities underscores the necessity for the development of alternative antimicrobial agents^2,3^. The current wound management strategies, which rely on conventional short-term antibiotics, may lose effectiveness and significantly impact clinical care^4–7^.

In addition to antibiotic employment, promoting the healing of acute wounds is a highly effective approach to preventing infection and chronic wounds. Utilizing tissue engineering techniques and advanced wound dressings is one of the most effective ways to accelerate wound healing. Since the cells can recognize the chemical and physical properties of their surrounding environment, numerous studies have been conducted on mimicking the composition and structure of the extracellular matrix (ECM) and its three-dimensional network^8,9^. Among the different structures utilized as advanced wound dressings, nanofibers are particularly notable due to their morphological similarity to ECM, high specific surface area and porosity, flexibility, permeability to oxygen, and the capability to incorporate active agents^10,11^. The natural ECM structure contains protein fibers with a diameter of 50–500 nm, which provide adhesion, growth, and the necessary biochemical microenvironment of cells^12^. Based on these characteristics, it is anticipated that nanofibrous wound dressings show improved cell behavior, such as enhanced cell adhesion and proliferation, compared to other types of wound dressings. The electrospinning method is a well-known, simple, and cost-effective technique for the production of nanofibers using a high-voltage electric field as the driving force^13–16^. Many researchers have used different electrospinning techniques to prepare antibacterial dressings. Wang et al.^17^ fabricated radially oriented berberine-loaded poly(3-hydroxybutyrateco-3-hydroxyvalerate) (PHBV) nanofibers using electrospinning technique. They found that a radially oriented pattern induces cell migration, and their results showed that high specific surface area of ​​nanofibers caused the effective release of berberine as well as anti-inflammatory and antibacterial activities. They also reported a 100% wound closure rate after 18 days of treating diabetic mouse full-thickness skin wounds with the fabricated nanofibers.

Diverse synthetic and natural biomaterials have been utilized in the development of wound dressings. Soy protein isolate (SPI), a natural polymer consisting of approximately 90% protein, has been widely used in different biomedical applications due to its abundant availability, cost-effectiveness, biocompatibility, and biodegradability. The structure of SPI comprises numerous amino acids (e.g., aspartic acid, glutamic acid, glycine, alanine, valine, leucine, lysine, and arginine) as well as a variety of reactive functional groups (e.g., amine, carboxyl, and hydroxyl), which significantly influence its biological behavior and its interactions with other materials and cells^18,19^. The intrinsic composition and properties of SPI have made it an effective and inexpensive substitute for growth factors^20^. Despite the numerous advantages, the poor mechanical properties of SPI, similar to many natural polymers, have made its blending with biocompatible synthetic polymers a preferred strategy over its pure utilization. Soluplus (Sol) is a graft-copolymer composed of polyvinyl caprolactam, polyvinyl acetate, and polyethylene glycol, which specifically developed to enhance the solubility of poorly soluble drugs (such as mupirocin) due to its amphiphilic properties^21^. Sol shows suitable plasticity and can be fabricated in various forms, including films and nanofibers^22,23^.

One of the essential properties of an ideal modern wound dressing is the ability to show antibacterial activity and prevent wound infections. Mupirocin (Mp) is an effective antibiotic agent that has been widely used against gram-positive and gram-negative bacteria and is particularly effective in the treatment of burn wounds infected with methicillin-resistant staphylococci. Furthermore, Mp is recognized not to interfere with the activity of other antibiotics^24,25^.

Based on the provided information, it is predicted that the fabrication of a Sol/SPI/Mp nanofibrous wound dressing with desirable characteristics, including great biocompatibility and cell adhesion, as well as appropriate antibacterial activity, has the potential to efficiently impede the infection of full-thickness skin wounds and accelerate the wound healing process. Other advantages of this wound dressing include mimicking the structure of ECM, proper swelling ability and absorption of exudate, which in addition to maintaining wound moisture, promotes cell adhesion and growth. To the best of our knowledge, the compound utilized in this nanofiber dressing has not been evaluated in any prior studies on skin tissue repair. In the current study, following the fabrication and characterization of the Sol/SPI/Mp nanofibrous dressing, comprehensive physical, mechanical, and biological studies were performed, and in vivo assessments were conducted on a rat model to investigate the properties of the prepared wound dressing.

## Materials and methods

### Materials

Sol (BASF, Germany), SPI (Balmy Life, Iran) and Mp (Pars Daru, Iran) were purchased to fabricate nanofibers. Formaldehyde 37%, methyl thiazole diphenyl tetrazolium bromide (MTT) powder, and dimethyl sulfoxide (DMSO) were obtained from Sigma-Aldrich, USA, and Dulbecco’s Modified Eagle Medium (DMEM) was obtained from Shanghai Yuan Biotechnology Co., China, for cell assessments. Injectable anesthetics, Ketamine and xylazine, were purchased from Rotex Media, Germany and from Alfasan, Netherlands, respectively. Human keratinocyte cells (HaCat) were also obtained from Institute Pasteur cell bank, Iran.

### Fabrication of nanofibrous dressings

Nanofiber dressings were fabricated through the electrospinning technique. Initially, a Sol solution with a concentration of 33% w/v was prepared in distilled water over 5 h at room temperature^23^. Concurrently, a 10% w/v SPI solution was prepared in distilled water^26^, and subsequently the Sol and SPI solutions were mixed in a ratio of 3:1. The Sol: SPI ratio was selected according to the morphology of electrospun nanofibers in pilot experiments. Figure S1 shows the morphology of nanofibers prepared with ratios of 1:1, 1:2, and 2:1, which had defected and beaded structures. The Sol/SPI/Mp solution was prepared by adding 2% w/w of Mp powder to the Sol solution before mixing with the SPI solution. Each of the Sol, Sol/SPI, and Sol/SPI/Mp solutions was poured separately into 5 mL plastic syringes with a 0.6 mm needle. The electrospinning process was conducted with a distance of 12 cm between the needle and the collector, a potential difference of 15 kV, and a feeding rate of 1 mL/h. This procedure resulted in the fabrication of Sol, Sol/SPI, and Sol/SPI/Mp nanofibrous dressings.

### Characterization

#### Morphological and physical assessments

A scanning electron microscopy (SEM; FEI Quanta-200, USA) at an accelerating voltage of 15 kV was used to visualize the morphology of the fabricated nanofibers. Before imaging, the samples were coated with a thin layer of gold. The diameters of the nanofibers (*n* = 40) were measured using ImageJ 1.52v software, and the results were presented as an average.

The specific surface area of ​​nanofibers was measured through the Brunauer-Emmett-Teller technique (BET; TPD9105; Iran). The adsorption/desorption isotherm of pure N_2_ gas was adjusted at a pressure of 0.88 atm and the amount of gas adsorbed on the nanofibers was measured by pressure-volume-temperature relationships.

To measure the porosity of Sol, Sol/SPI and Sol/SPI/Mp nanofibers using the liquid displacement method, first, the nanofibrous pieces with dimensions of 10 × 10 mm were weighed and immersed in 5 mL of distilled water, and then the porosity was according to Eq. (1):

1 $$\:Porosity\:\left(\%\right)=\:\frac{{V}_{1}-\:{V}_{3}}{{V}_{2}-\:{V}_{3}}$$

V1, V2, and V3 are the initial volume of water, the volume of water during immersion, and the volume of remaining water after removing of nanofibers, respectively.

#### Investigation of functional groups

The evaluation of chemical composition and functional groups of the fabricated nanofibers was performed through Fourier transform infrared spectroscopy (FTIR; Bruker, Tensor 2, Germany). For this purpose, FTIR spectra of the Sol/SPI and Sol/SPI/Mp nanofibers were obtained within the range of 400–4000 cm^−1^ wavelength, and a resolution of 4 cm^−1^ with 40 scans.

#### Evaluation of water absorption and swelling

The water absorption and swelling of the electrospun nanofibers were examined via immersion in distilled water. Nanofibers with dimensions of 20 × 20 mm were immersed in 50 mL of distilled water, and after 24 h, the samples were removed and weighed. The water absorption capacity was measured by Eq. (2) ^27,28^:

2 $$\:Water\:absorption\:\left(\%\right)=\:\frac{{W}_{w}-\:{W}_{d}}{{W}_{d}}\:\times\:100$$

In this equation, W_d_ represents the weight of dry nanofiber sample, while W_w_ represents the weight of swollen nanofiber sample.

#### Water vapor permeability

The water vapor permeability (WVP) of the electrospun nanofibers was assessed based on the standard method (ASTM E96-00)^29,30^. Briefly, the nanofiber wound dressings were placed on the rim of a 25 mL beaker containing 10 mL of water and sealed at the edges with parafilm. The beakers were then weighed and incubated at a temperature of 37 °C for 24 h. The WVP was measured using Eq. (3):

3 $${\text{WVP (g/m}}^{2} {\text{day)}} = \frac{\varDelta\:W}{A.t}$$

where ΔW represents the change in the weight of the beaker, A is the area of the beaker rim (exposed surface), and t is the test duration (one day).

### Biodegradability

The weight loss of nanofibrous wound dressings after immersion in phosphate-buffered saline (PBS) solution was evaluated as in-vitro biodegradability according to the ASTM F1635-11 standard method^31^. Initially, wound dressing with dimensions of 10 × 10 mm was weighed and then incubated in 10 mL of PBS solution at 37 °C. On days 1, 3 and 5, the nanofibers were removed and freeze-dried to assess the dry mass post degradation. Equation (4) was applied to measure the biodegradability.

4 $$\:Biodegradability\left(\%\right)=\:\frac{{W}_{0}-\:{W}_{t}}{{W}_{0}}\:\times\:100$$

In this equation, W_0_ denotes the initial weight of the wound dressing and W_t_ shows the weight after degradation.

### Mechanical properties

Tensile properties, including tensile strength and tensile modulus of nanofibrous wound dressings with dimensions of 10 × 30 mm were determined according to ASTM-D882 by a Zwick-Germany testing machine^32^. The tensile test was performed at a rate of 5 mm/min using a 50 N load cell and at a temperature of 25 ± 1 °C. Finally, the information related to the tensile properties was collected from the stress-strain diagram and analyzed.

### Drug release

The release profile of Mp from Sol/SPI/Mp nanofibrous wound dressing was evaluated in an in-vitro study. A wound dressing sample (40 × 40 mm) was incubated in a container with 60 mL of PBS solution. Subsequently, at 1, 12, 24, 48, 72, 96, and 120 h time points, 3 mL of the PBS solution containing the released Mp was extracted with a syringe, and 3 mL of fresh medium was replenished. The concentration of released Mp was then quantified using UV spectrophotometry at a wavelength of 221 nm^33^.

### Antibacterial activity

The antibacterial effectiveness of electrospun nanofibrous wound dressings was assessed against Staphylococcus aureus (S. aureus), a gram-positive bacterium, and Escherichia coli (E. coli), a gram-negative bacterium, following the method described by Rafieerad et al.^34^. Nanofibers with a dimension of 1 cm^2^, were placed in the wells of a 24-well culture plate, and 1 mL of bacterial suspension containing 10^8^ CFU/mL was added to them. The samples were then incubated for 24 h. In the next step, the viable bacteria were counted through the serial dilution method after subjecting the samples to 10 min of ultrasonic vibration. The antibacterial activity was calculated using Eq. (5):

5 $$\:Antibacterial\:activity\:\left(\%\right)=\:\frac{B-A}{B}\:\times\:100$$

where B is the number of viable bacteria in the control culture medium (well without nanofiber) and A is the number of viable bacteria in the culture medium of the well containing nanofiber.

### Hemolysis assay

Hemolysis assay was carried out to evaluate the blood compatibility of the fabricated nanofibers according to ASTM F756. Briefly, the collected blood was poured into EDTA vials and centrifuged at 2000 rpm for 8 min and the supernatant was discarded. The obtained RBC pellet was dissolved in PBS with a ratio of 1:9 using a magnetic stirrer at room temperature. The nanofibers (10 × 10 mm) were submerged in normal saline at 37 °C for 30 min and then placed in a 24-well plate containing 1 mL of diluted RBC solution. After 1 h incubation at 37 °C, the absorbance of the supernatant was recorded at 540 nm, and hemolysis was calculated according to Eq. (6). In this assessment, 1% Triton X (100 µL) was considered as a positive control (100% hemolysis) and PBS (100 µL) as a negative control (0% hemolysis).

6 $$\:Hemolysis\:\left(\%\right)=\:\frac{{A}_{s}-\:{A}_{n}}{{A}_{p}-\:{A}_{n}}\:\times\:100$$

In this equation, the absorption of sample, positive control, and negative control were represented by A_s_, A_p_, and A_n_, respectively.

### In vitro cellular assessments

Human keratinocyte cells (HaCat, Pasteur Institute Cell Bank, Iran) were used to evaluate the biocompatibility of electrospun nanofibers. The cells were cultured in DMEM supplemented with 10% FBS, 1% penicillin (100 U/mL), 1% streptomycin (100 µg/mL), and 1% amphotericin B (250 µg/mL) under standard cell culture conditions. Upon reaching 80% confluency, the HaCaT cells were trypsinized, separated by centrifugation, and prepared for MTT and cell adhesion assays. The viability of HaCat cells on nanofibrous wound dressings was assessed using the MTT test based on metabolic activity. Initially, 20,000 HaCat cells were seeded on 10 × 10 mm nanofibers in a 24-well culture plate and the well without any nanofiber was served as the control sample. After 1 and 5 days of culture, the supernatant of each well was replaced with 50 µL of MTT solution (0.5 mg/mL). Following a 4-h incubation period, formazan crystals were dissolved in 200 µL of DMSO per well, and the optical density (OD) was measured at 570 nm using an Eliza Reader^35^. The cell viability was then calculated using Eq. (7).

7 $$\:Cell\:viability\left(\%\right)=\:\frac{\text{O}\text{D}\:\text{o}\text{f}\:\text{s}\text{a}\text{m}\text{p}\text{l}\text{e}\:\:}{\text{O}\text{D}\:\text{o}\text{f}\:\text{c}\text{o}\text{n}\text{t}\text{r}\text{o}\text{l}}\:\times\:100$$

To evaluate cell adhesion and morphology, HaCat cells were cultured on nanofibers for 2 days, as described. Subsequently, the cell-seeded nanofibrous samples were fixed with 3% glutaraldehyde, dehydrated using a series of increasing ethanol concentrations, and subjected to SEM imaging.

### Skin irritation test

Skin irritation test was carried out for Sol, Sol/SPI and Sol/SPI/Mp nanofibers, according to ISO10993-10 ^36,37^ using three healthy male Wistar rats for each group. The dorsal hair of the animals was shaved and 10 × 10 mm nanofibrous samples were applied to the back of each rat. The nanofibers were stabilized with sterile gauze and hypoallergenic adhesive at the desired location. The rats in each group were examined for skin irritation and allergic reactions after 24, 48, and 72 h.

### In vivo animal evaluation

To investigate the wound healing efficacy of the electrospun nanofibrous wound dressings, a full-thickness wound model was utilized in Wistar rats (animal nest of Isfahan University of Medical Sciences), aged 4 months and weighing 300 ± 20 g. All animal experiment procedures were in accordance with EU Directive 2010/ 63/EU and ARRIVE guidelines and the animal studies were performed after the approval of the ethics committee of Isfahan University of Medical Sciences, Iran (ethics code #IR.MUI.AEC.1402.020). The animals were housed under standard experimental conditions with free access to food and water, and a 12-h light/dark cycle. Twenty-four rats were randomly divided into four groups including blank (no wound dressing), Sol, Sol/SPI, and Sol/SPI/Mp.

Prior to the surgical procedure, a combination of ketamine (80 mg/kg) and xylazine (10 mg/kg) was injected intraperitoneally to induce general anesthesia. After shaving the dorsal area of the animals, the surgical area was disinfected using 70% ethanol, followed by the application of betadine solution. A full-thickness circular wound with a 10 mm diameter was created by a biopsy punch on each rat. The wound area in the treatment groups was covered with nanofiber dressing and stabilized by a secondary dressing (sterile gauze and hypoallergenic adhesive). In the control group, the wound surface remained uncovered without any nanofibrous dressing. The animals were then returned to their standard conditions and the wound dressing changed every two days after washing the wound site with sterile normal saline.

To evaluate the wound closure progress, the surface of the wounds was imaged on days 1, 7, and 14, post-surgery. On day 14, the animals were euthanized by carbon dioxide inhalation and the wound tissue, along with the adjacent skin, was surgically excised. The harvested tissue samples were fixed in 10% formaldehyde solution overnight. Paraffin-embedded tissue sections with a thickness of 4 μm were prepared and stained with hematoxylin and eosin (H&E) and Masson’s trichrome for histopathological analysis. Moreover, immunohistochemical evaluations were performed by detecting the expression of cluster of differentiation-31 and 34 (CD-31 and CD-34), then examined under an optical microscope.

### Statistical analysis

All the tests were performed with three repetitions for each experimental group, and the numerical data are reported as mean ± standard deviation. The one-way ANOVA test was carried out for statistical evaluation of the data. In this study, *p* < 0.05 was considered statistically significant, and *, **, ***, and **** represented *p* < 0.05, *p* < 0.01, *p* < 0.001, and *p* < 0.0001, respectively.

## Results and discussion

### Morphological and physical assessments

**Fig. 1:**
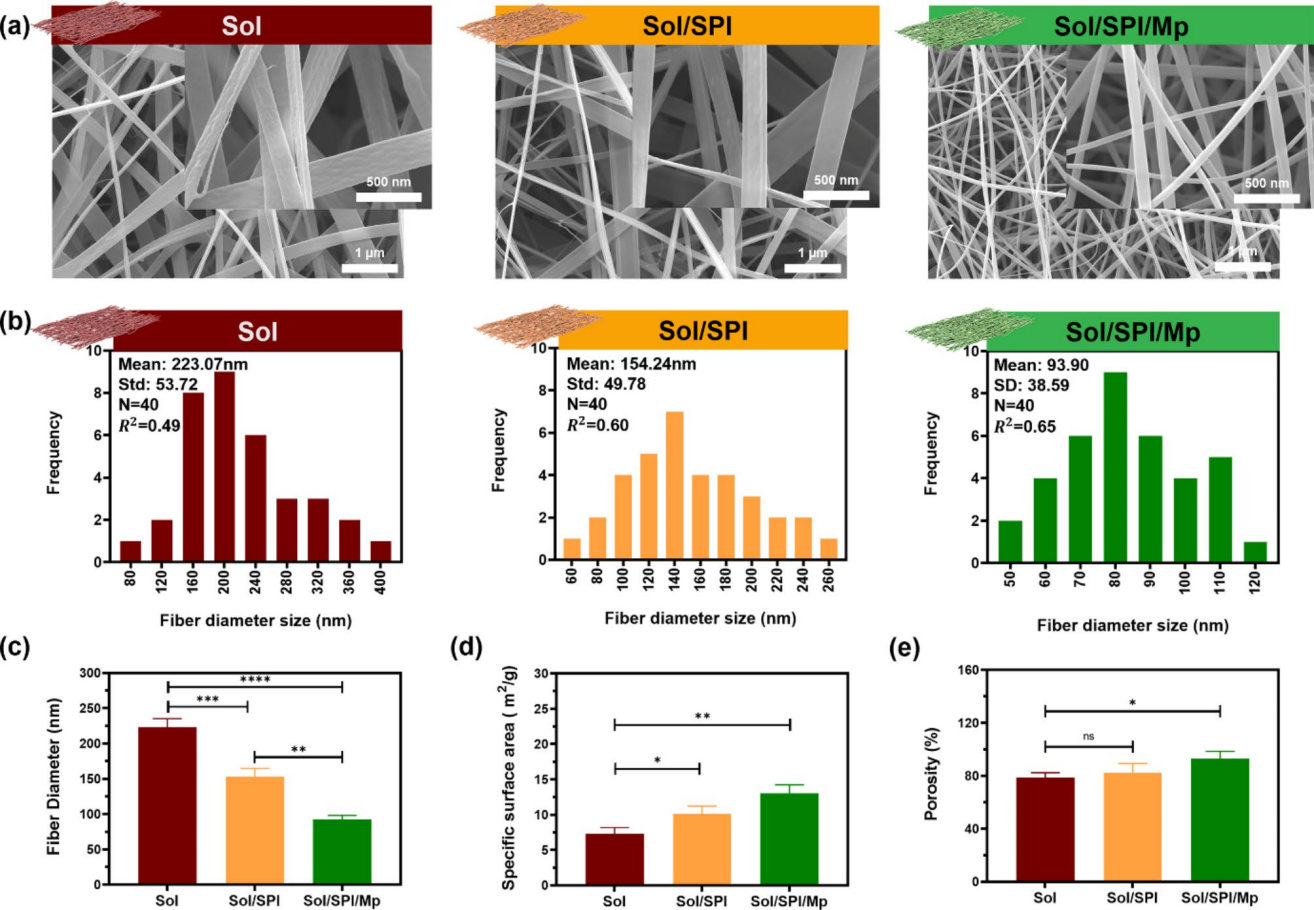
(**a**) SEM images of Sol, Sol/SPI and Sol/SPI/Mp nanofibers, (**b**) the diagrams of diameter distribution related to different nanofibers, (**c**) fiber diameter bar graph, (**d**) specific surface area bar graph, and (**e**) porosity bar graph.

Due to the similarity with ECM, nanofibrous structures are among the most desirable constructs for the fabrication of wound dressing. The SEM images of the electrospun nanofibers developed in this study are presented in Fig. 1a. The SEM micrographs reveal that all the fiber structures were continuously electrospun without any observable defects, and the primary difference between the various nanofibers was in their diameter. The average diameter of the Sol nanofibers was measured as 223.07 ± 53.72 nm, while the incorporation of SPI and SPI/Mp led to a decrease in the average nanofiber diameter to 154.24 ± 49.78 nm and 93.90 ± 38.59 nm, respectively (Fig. 1b,c). This reduction in the nanofiber diameter can be attributed to the increased viscosity, improved intermolecular interactions between the polymer chains, and the enhanced elasticity of the electrospun jet in the electric field^38^.

The specific surface area of ​​Sol, Sol/SPI and Sol/SPI/Mp nanofibers was measured using the BET test and its results are given in Fig. 1d. As mentioned, with the addition of SPI and Mp, the diameter of Sol nanofibers decreased dramatically. This reduction in the diameter of nanofibers finally led to an increase in the specific surface area, so that the highest specific surface area was related to Sol/SPI/Mp (13.03 ± 1.19 m^2^/g) nanofibers. Increasing the specific surface area leads to better cell-nanofiber interaction and can effectively accelerate wound healing.

Also, exudate absorption capacity, water vapor permeability, and cellular ingrowth are among the characteristics that are highly dependent on the porosity of a wound dressing^39^. The porosity of the fabricated nanofibers was measured through the liquid displacement method (Fig. 1e). All electrospun nanofibers exhibited high porosity levels, and the porosity of Sol, Sol/SPI, and Sol/SPI/Mp nanofibers was measured as 78.53 ± 3.85, 82.50 ± 6.78, and 92.93 ± 5.59%, respectively. The decrease in the diameter of nanofibers was the main reason for increasing porosity in Sol/SPI/Mp nanofibers compared to Sol and Sol/SPI nanofibers.

### Investigation of functional groups

**Fig. 2:**
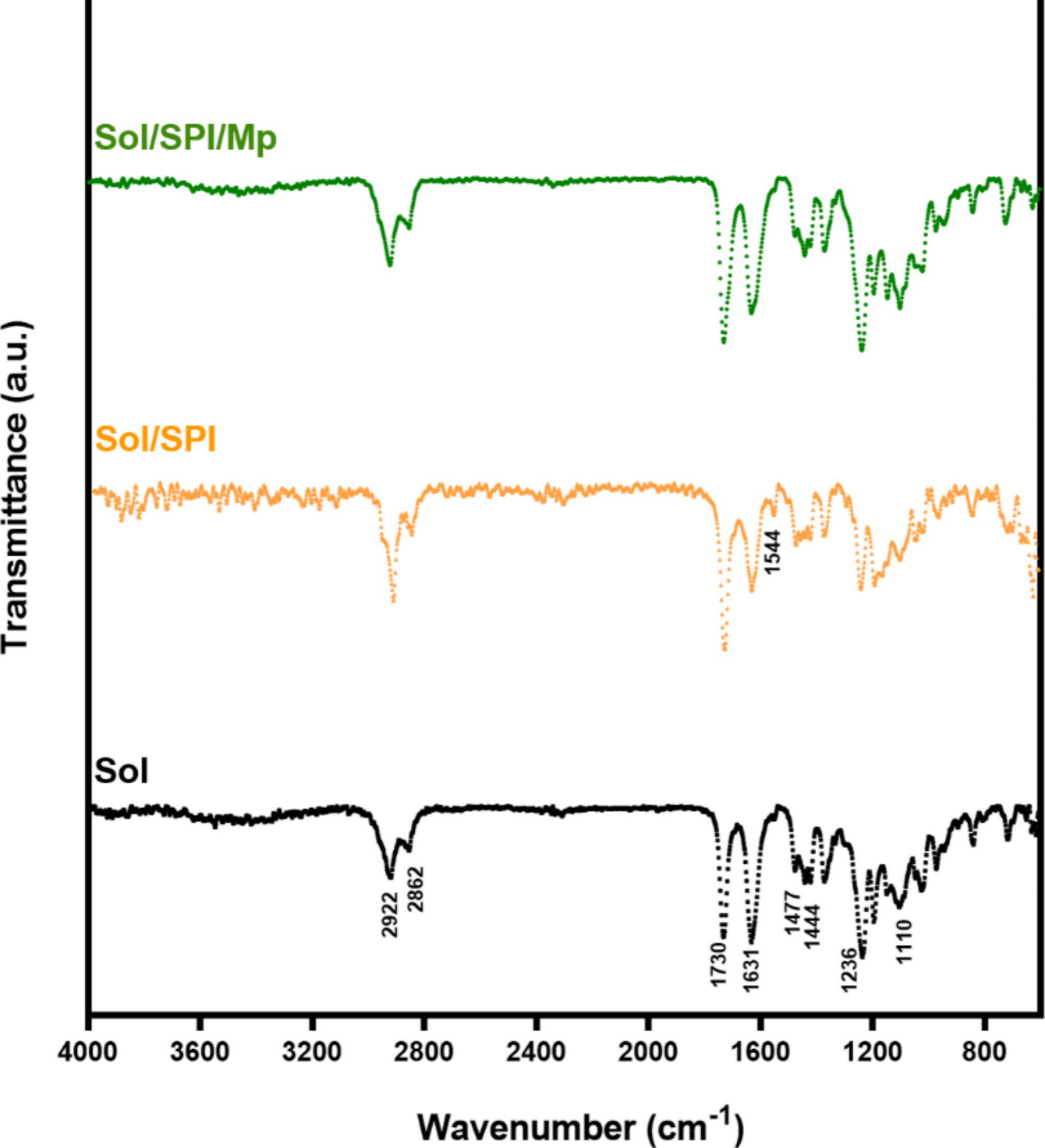
FTIR spectra of pure Sol, Sol/SPI and Sol/SPI/Mp nanofibers.

The IR spectra of different samples are displayed in Fig. 2. The symmetric and asymmetric stretching of CH appeared at 2862 and 2922 cm^−1^, respectively. Two distinct bands at 1631 and 1730 cm^−1^ are correspondingly related to amide and ester carbonyl groups in the Sol structure. The bands at 1444 and 1477 cm^−1^ are related to CH3 and C–O–C bending vibrations, respectively, while ester C–O stretching vibrations can be detected at 1236 and 1110 cm^−1^ bands^40^. In the Sol/SPI spectrum, a new peak has appeared at 1554 cm^−1^, which is related to amide II in the SPI structure. Amide I can also be detected at the wavelength of 1631 cm^−1^, the intensity of which has decreased compared to the pure Sol spectrum, due to the hydrogen interactions between Sol and SPI functional groups^41^. In the Sol/SPI/Mp spectrum, all the index peaks of the constituent components with changed intensities can be identified. The presence of Mp is detected by the characteristic peaks at 1730 and 1631 cm^−1^, which are attributed to the stretching vibrations of the C=O group, however, due to the overlap of the bands and the low amount of Mp in the composition, only the peaks at 1730 and 1631 cm^−1^ were intensified compared to the Sol/SPI spectrum.

### Assessment of water absorption and swelling

**Fig. 3:**
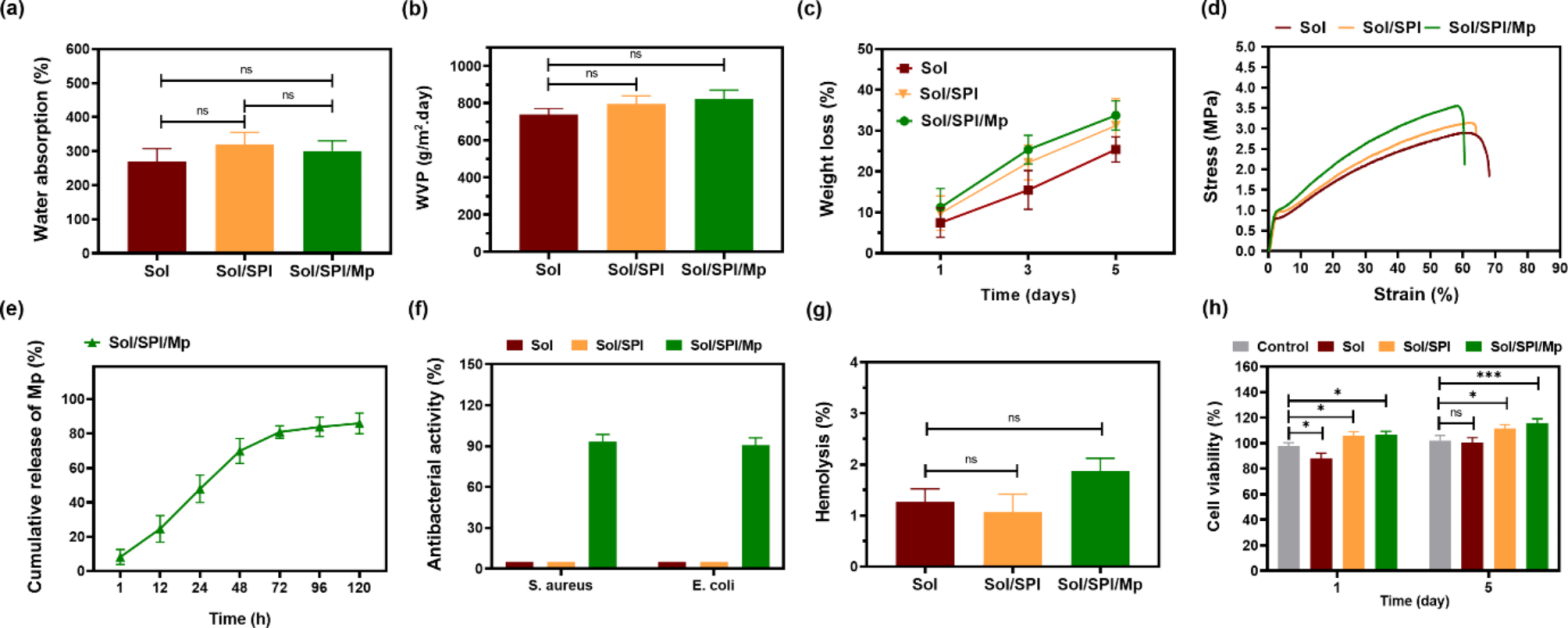
Diagrams of (**a**) water absorption, (**b**) WVP, (**c**) degradability in PBS, (**d**) tensile stress-strain, (**e**) Mp release profile, (**f**) antibacterial activity against S. aureus and E. coli, (**g**) hemolysis ratio, and (**h**) cell viability of the electrospun nanofibers.

Severe and full-thickness wounds are often characterized by the presence of abundant wound secretions. An ideal wound dressing should possess the ability to effectively absorb the exudate and prevent infection. Simultaneously, the dressing should also help maintain the optimal moisture level at the wound surface and prevent the scab formation^42^. Furthermore, preventing dehydration and maintaining the moisture of the wound surface improves re-epithelialization process and wound contraction^43^. The water absorption and swelling behavior of the electrospun nanofiber wound dressings were evaluated after 24 h of immersion in distilled water, as shown in Fig. 3a. The nanofiber dressings exhibited a significant degree of swelling. Indeed, the water absorption of Sol, Sol/SPI, and Sol/SPI/Mp nanofibers was measured as 270.0 ± 37.5, 319.5 ± 35.7, and 300.8 ± 29.7%, respectively. However, the differences in the water absorption capacity among the various nanofibers were not statistically significant (*p* > 0.05). This water absorption and the subsequent swelling can be attributed to the presence of abundant hydrophilic functional groups, such as hydroxyl and amide groups, within the polymer structures that comprise the nanofibers.

### Water vapor permeability

The permeability of a wound dressing to water vapor is a crucial property, as it can significantly impact the wound healing process. Inadequate permeability can lead to the accumulation of wound exudates and an increased risk of infection, while excessive permeability may cause wound dehydration and scar formation^44^. The ability of nanofibers to pass the water vapor was evaluated and it was demonstrated effective permeability in all three types of nanofibrous dressings (Fig. 3b). The WVP values for the Sol, Sol/SPI and Sol/SPI/Mp nanofibrous dressings were measured as 739.1 ± 31.8, 795.0 ± 45.0 and 821.8 ± 49.1 g/m^2^ day (*p* > 0.05). Since, the reported rates of water evaporation from the surface of fresh wounds are in the range of 279 to 5138 g/m^2^ day^45^, therefore, the WVP of the electrospun nanofibrous dressings falls within the standard range.

### Biodegradability

The replacement of the newly formed tissue with a porous matrix used as a tissue engineering scaffold or wound dressing, is achievable through the degradation of the biomaterial at a suitable rate. The weight loss graph of the nanofibrous wound dressings over time is shown in Fig. 3c. All three wound dressings exhibited a notable but controlled weight loss up to day 5. The Sol, Sol/SPI and Sol/SPI/Mp nanofibers displayed weight loss of 25.40 ± 3.10, 31.23 ± 6.58, and 33.73 ± 3.55%, respectively, after 5 days. The presence of SPI with the higher concentration of hydrolysis-sensitive groups in the composition of nanofibers accelerated the degradation rate^46^. In a similar study, Varshney et al.^47^ stated that the addition of silk fibroin controlled the inferior hydrolytic stability of SPI nanofibers and reduced the biodegradation rate. Here, the blending of Sol as a synthetic polymer with SPI controlled the biodegradation of nanofibers. Also, the results indicated that the hydrolysis of the polymer chains was inversely proportional to the diameter of the nanofibers. By reducing the nanofiber diameter and increasing the specific surface area, the degradability of the wound dressings was enhanced.

### Mechanical properties

The stress-strain diagram of the nanofibers under tensile loading is presented in Fig. 3d, and the corresponding mechanical properties are summarized in Table 1 and Fig. S2. The Sol nanofibers exhibited the lowest ultimate stress and the highest elongation at break among the samples. However, the inclusion of SPI and Mp into the nanofibers led to an increase in the ultimate stress and a decrease in the elongation at break. The Sol/SPI/Mp nanofibers revealed the highest ultimate stress (3.61 ± 0.29 MPa) and the highest elastic modulus (39.15 ± 5.08 MPa) accompanied by the lowest elongation at break (59.11 ± 1.94%). According to the results, decreasing the diameter of nanofibers enhanced their mechanical strength. By reducing the diameter of the electrospun jet and produced nanofibers in the electrospinning process, the molecular orientation of the polymer solution becomes more regular, and the crystallinity of the nanofibers increases. This structural transformation leads to enhanced tensile strength and elastic modulus of the nanofibers, while reducing ductility^48^. Studies have reported that tensile strength, elastic modulus, and elongation at break of human skin are in the range of 1–32 MPa, 4–83 MPa, and 17–207%, respectively^49–51^. Considering this matter, the nanofibers developed in this study as temporary wound dressings can exhibit satisfactory performance in terms of their mechanical properties.

**Table 1:** Mechanical properties of the nanofibers under tensile stress.

Sample	Ultimate stress (MPa)	Elastic modulus (MPa)	Elongation at break (%)
Sol	2.73 ± 0.14	34.40 ± 4.32	66.24 ± 3.65
Sol/SPI	3.19 ± 0.21	35.86 ± 3.73	63.28 ± 2.12
Sol/SPI/Mp	3.61 ± 0.29**	39.15 ± 5.08	59.11 ± 1.94*

* and ** indicate statistically significant differences with Sol sample and represent *p* < 0.05 and *p* < 0.01, respectively.

### Drug release

The release profile of Mp from the Sol/SPI-Mp wound dressing was evaluated in the PBS solution during 5 days, as shown in Fig. 3e. Initially, the release occurred at a relatively slow rate, with about 8.20 ± 4.37% of Mp being released during the first hour. This was followed by a burst release phase, with approximately 69.86 ± 7.20% of Mp being released by the 48th hour. Following this, the release rate slowed down from 48 to 120 h, and by the 120th h, a total of 85.90 ± 6.02% of the loaded Mp had been released. The two key factors which influenced the release of Mp were the swelling and the degradation of the nanofibers. Initially, when the nanofibers were immersed in the PBS solution, there was a delay for the nanofibers to reach their equilibrium swelling state and the increased swelling led to the observed burst release of Mp. Consequently, swelling constituted the predominant factor in the release process up to 48 h. Once equilibrium swelling was achieved, the release rate slowed down. It became evident that the degradability emerged as the predominant factor, influencing the release from 48 to 120 h.

### Antibacterial activity

Any acute skin injury is susceptible to microbial infection and develops into chronic wounds. Therefore, protecting the wound and preventing the invasion and proliferation of microorganisms during treatment is a crucial aspect in the management of acute skin injuries. The antibacterial efficacy of electrospun nanofibrous dressings was evaluated against S. aureus and E. coli, as depicted in Fig. 3f. The findings showed that the Sol and Sol/SPI nanofibers did not exhibit any significant antibacterial properties during the incubation period. In contrast, the Sol/SPI/Mp nanofibrous dressing demonstrated excellent performance, achieving more than 90% effectiveness against both bacterial strains. Based on the results, the release of Mp from the Sol/SPI/Mp nanofibrous dressing was the primary factor responsible for the observed antibacterial efficacy. Several previous studies have reported the potent antibacterial and anti-biofilm formation activity of Mp against E. coli and S. aureus bacteria^52–54^. Indeed, Mp causes effective antibacterial activity by inhibiting bacterial protein synthesis by specific reversible binding to bacterial isoleucyl tRNA synthase^55^.

### Hemolysis assay and in vitro cellular assessments

In order to investigate hemocompatibility of electrospun nanofibers, the hemolysis assay was carried out and its results are shown in Fig. 3g. In this test, the release of hemoglobin as an indicator of erythrocyte membrane disruption is calculated^56^. The standard hemolysis ratio for blood compatible products has been reported to be less than 5% in some studies^57^ and less than 2% in others^56^. Since the hemolysis ratio for all electrospun nanofibers was less than 2%, these samples can be classified as non-hemolytic materials.

Keratinocytes are structural cells of skin tissue, which can even act as key immune cells^58^, therefore, cellular studies were performed on a human keratinocyte cell line (HaCat). According to Fig. 3h, all the prepared wound dressings exhibited good compatibility with HaCat cells, and the cell viability was higher than 80% which showed that none of the samples induced cytotoxicity^59^. On day 1, the cell viability on the Sol nanofibrous dressing was significantly lower than the control sample (*p* < 0.05). In contrast, the Sol/SPI nanofibers showed higher cell viability compared to the control on both days 1 and 5 (*p* < 0.05). Furthermore, the proliferation of HaCat cells on the Sol/SPI/Mp nanofibrous dressing was the highest, and this sample exhibited the highest cell viability on day 5 (*p* < 0.001).

**Fig. 4:**
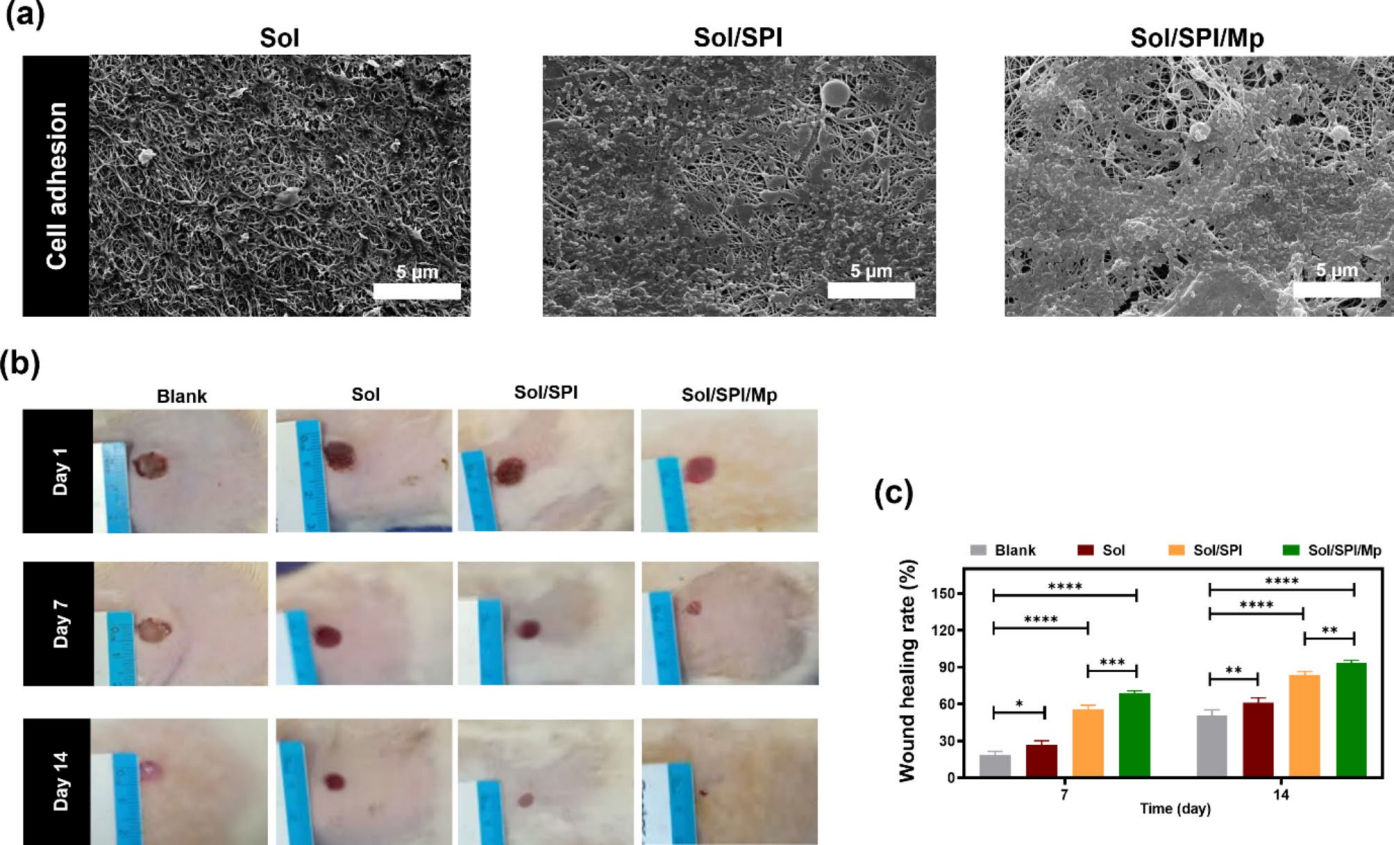
(**a**) Adhesion of HaCat cells on the nanofibers, (**b**) images of the wound surfaces on days 1, 7 and 14, (**c**) wound closure diagram.

Consistent with the results of the MTT assay, the results from Fig. 4a illustrated that the cell adhesion on the Sol wound dressing was limited, with the presence of spherical cells sparsely distributed across the surface. However, the incorporation of SPI into the nanofibers led to an increase in the density of attached cells on the surface. Notably, the cell adhesion, spreading, and density were remarkably higher on the nanofibers containing Mp compared to the other samples. Additionally, these cells appeared to secrete ECM-like structures.

The success of a matrix used in tissue engineering is directly correlated with its ability to mimic the biological environment and ECM which leads to enhanced cell behavior. Notably, electrospun nanofibers are recognized as one of the most biomimetic structures that possesses an ECM-like architecture. The properties of electrospun nanofibers, including their reduced diameter and increased specific surface area, improve the cell-matrix interactions^60,61^. The presence of SPI in the Sol/SPI and Sol/SPI/Mp wound dressings significantly influenced cell proliferation and adhesion due to the presence of peptides similar to the ECM^20,62,63^. However, the Sol/SPI/Mp nanofibers exhibited the highest cell compatibility, possibly attributed to the release of Mp. Several previous studies have reported that Mp can improve cell behavior by stimulating the secretion of endogenous growth factors^33,64^.

### Skin irritation test

To determine the irritant properties of Sol, Sol/SPI and Sol/SPI/Mp nanofibers, skin irritation test was performed and the score of irritation (erythema and edema) was selected for each group according to ISO10993-10 ^37^. The score of erythema and edema after application of the nanofibers is summarized in Table S1. Based on observations, there were no erythema and edema effects on the test site in any of the rats, and the nanofibers did not show any skin irritation during 72 h of the usage.

### In vivo animal evaluation

The animal study was conducted for a duration of 14 days, and the wound closure in the treated groups was compared to the blank (control) group, as shown in Fig. 4b,c. The wound closure for the blank group on days 7 and 14 was 18.50 ± 2.90 and 50.93 ± 4.35%, respectively. The pure Sol nanofibers on the wound surface significantly enhanced wound healing and the wound closure was improved by 26.86 ± 3.36 on day 7 and 61.10 ± 4.01% on day 14 compared to the control (*p* < 0.05 and *p* < 0.01, respectively). The healing efficiency in the groups treated with the Sol/SPI and Sol/SPI/Mp nanofibrous dressings was substantially superior to the Sol and control groups. Treatment with the Sol/SPI and Sol/SPI/Mp nanofibrous dressings resulted in 83.36 ± 3.15 and 93.20 ± 2.45% wound closure after 14 days, respectively. Also, there were no signs of scar formation in these groups, which can be important from both functional and cosmetic points of view^65^.

**Fig. 5:**
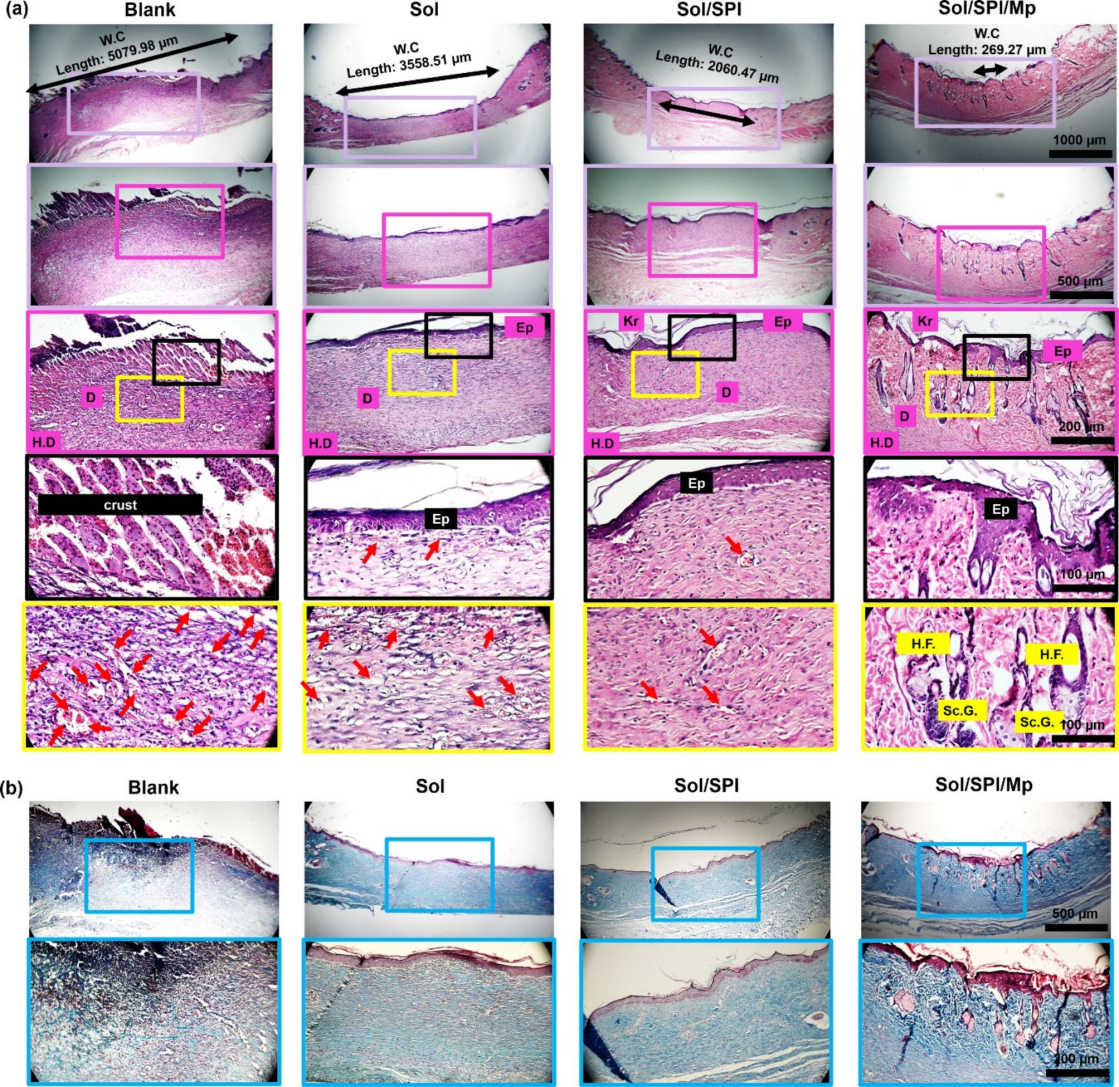
Histopathological images (with different magnifications) related to the tissue sections of the Blank group and the treated groups stained with (**a**) H&E and (**b**) Masson’s trichrome.

Figure 5a illustrate the histopathological sections stained with H&E for the Blank group, as well as Sol, Sol/SPI, and Sol/SPI/Mp treatment groups after 14 days. The key histological features, including the keratin (Kr) layer, regenerated epidermis (E), dermis (D), and hypodermis (H.D), are marked on the images for each treatment group. In the Blank group, there was no evidence of the E layer formation, and a wound crust was clearly visible. Conversely, in the groups treated with the Sol, Sol/SPI, and Sol/SPI/Mp nanofibrous dressings, the thickness of the regenerated E layer increased, and the Kr layer was well observed. Specifically, the highest degree of E and Kr layer formation was observed in the group treated with the Sol/SPI/Mp nanofibrous dressing. Also, the red arrows indicate the areas of neovascularization at the beginning stages of the repair process, which in the treated groups, especially Sol/SPI/Mp, are less than the control group. Considering the wound closure (W.C) based on the presence and growth of hair follicles on both sides of the skin (right and left area), the size of W.C was measured for the Blank, Sol, Sol/SPI, and Sol/SPI/Mp groups as 5079.98 μm, 3558.51 μm, 2060.47 μm, and 269.27 μm, respectively. Therefore, the Sol/SPI/Mp treatment group underwent the wound healing process at a faster rate. Furthermore, the presence of skin appendages, such as hair follicles (H.F.) and sebaceous glands (Sc.G.), in this group signifies the final stages of wound repair.

According to Fig. 5b, collagen deposition and maturation in the tissue sections of different groups stained with Masson’s trichrome is observed in blue color. The thickness of collagen bundles in the repaired areas in the group treated with Sol/SPI/Mp wound dressing is higher than in the Sol and Sol/SPI groups. It is worth noting that the deposition of collagen is seen in a disorganized manner in the Blank group.

**Fig. 6:**
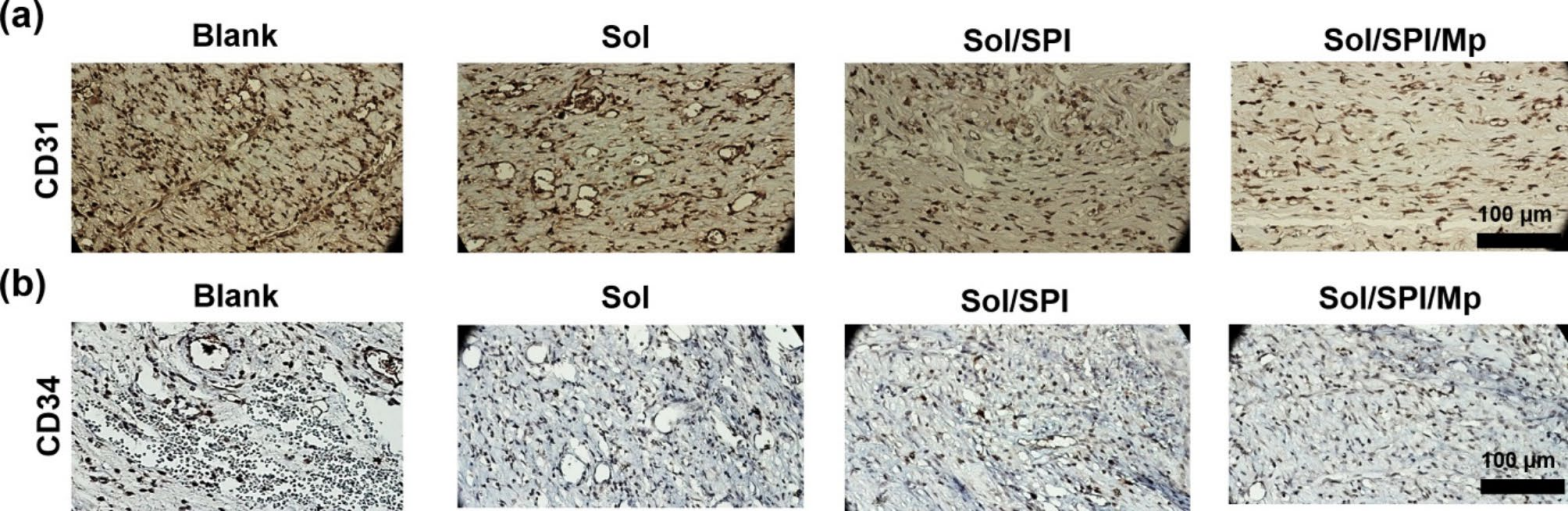
the results of immuno-histochemical analysis after 14 days: (**a**) CD31- and (**b**) CD34-stained tissue sections.

The delivery of oxygen and nutrients to the regenerating tissue and remove catabolic waste products is carried out through the capillary network formed during angiogenesis, which is one of the most important early stages of the repair process. As the healing process progresses, this newly formed capillary network gradually undergoes regression so that the vessel density becomes similar to that of healthy tissue^66^. Therefore, one of the ways to investigate wound healing is to examine angiogenesis in the regenerating tissue. Immunohistochemical studies of tissue sections were performed by examining the expression of CD31 and CD34 (Fig. 6a,b). Small arteries, arterioles, venules, and capillaries in the tissue sections are CD-31 and CD-34 positive, which can be seen in brown. As can be seen, the number of these positive points in the Blank group is more than the treatment groups. This indicates that the regenerating tissue in the Blank group was rich in capillary networks formed at the beginning of the healing process, and in fact, wound healing was in the initial stages after 14 days. In particular, the lowest density of CD31 and CD34 positive points are seen in tissue sections of Sol/SPI/Mp group, which indicates perfect wound healing after 14 days.

Gerstenhaber et al.^67^ prepared an electrospun soy protein-based dressing for the treatment of a pig model full thickness excisional wound. Based on their histopathological studies, robust signs of reepithelialization were observed in the group treated with SPI-based nanofibers. In this group, the epithelial layer in the epidermis was completely formed after 4 weeks, and the dermis had a completely normal and organized appearance. Also, some features such as the reduction of inflammatory cells, organized collagen bundles, and the presence of dermal appendages, were all signs of effective repair by SPI-based nanofibers. The extraordinary effect of SPI on in vivo wound healing has been stated in many other studies^68–70^.

Here, the fabricated nanofibrous wound dressings, with their ECM-like structure, maintained the moisture of the wound surface effectively, provided WVP at the standard level, and promoted a suitable environment for cell adhesion and proliferation. Consequently, the wound healing process was accelerated in the treated groups compared to the control group. Furthermore, the inclusion of SPI and Mp as bioactive agents in the Sol/SPI and Sol/SPI/Mp nanofibrous dressings optimized the cell behavior, which resulted in an even faster and more effective wound repair in these treatment groups^64,71^.

## Conclusion

The main focus of this study was on the fabrication and characterization of the Sol/SPI/Mp nanofibrous wound dressing. After analyzing physical, morphological, and chemical properties, the nanofibers degradability in PBS solution and their tensile mechanical properties were assessed. Thereafter, the release of Mp from the nanofibrous wound dressing was monitored over a 5-day period. The results showed that among all the fabricated wound dressings, only the nanofibrous dressing containing Mp exhibited the ability to effectively kill bacteria. The cell studies revealed that the highest viability of HaCat cells, as well as the greatest cell adhesion and spreading, were observed on the Sol/SPI/Mp nanofibers. Additionally, the in vivo experiments displayed that the group treated with the Sol/SPI/Mp nanofibrous wound dressing achieved the highest rate of W.C and re-epithelialization during 14 days. According to the results of this study, the produced electrospun nanofibrous dressing is recommended for use in the repair of full-thickness wounds with a risk of infection. The non-use of rats with infected wounds in animal studies, the low thickness of electrospun nanofibrous membranes for use in deep wounds, and the need to use secondary dressings to stabilize the nanofibers at the wound site are among the weaknesses of the present study. Although the promising results obtained from the in vitro and in vivo studies are encouraging, it is necessary to conduct clinical studies to further validate and confirm the efficacy of this nanofibrous wound dressing in a clinical setting.

## Electronic supplementary material

Below is the link to the electronic supplementary material.

Supplementary Material 1

## Data Availability

All data generated or analysed during this study are included in this published article.
