# Supplementary material for: Antibacterial and wound healing stimulant nanofibrous dressing consisting of soluplus and soy protein isolate loaded with mupirocin
Source: Sci Rep. 2024 Nov 2;14:26397. doi: 10.1038/s41598-024-78161-4 (PMC11531482; doi:10.1038/s41598-024-78161-4)
Supplement: Supplementary file 1 — Supplementary Material 1 [file 41598_2024_78161_MOESM1_ESM.docx]

**Supporting file**

**Journal:** Nature Scientific Reports

**Manuscript ID:** Submission ID 4df5584d-0495-46dc-8504-1406a4aa482f **Title:**  Antibacterial and Wound Healing Stimulant Nanofibrous Dressing Consisting of Soluplus and Soy Protein Isolate Loaded with Mupirocin

**Authours:** Maryam Jahani, Azadeh Asefnejad, Mastafa H. Al-Musawi, Ahmed A. Mohammed, Basma Talib Al-Sudani, Maha Hameed Al-bahrani, Nada A.Kadhim, Mina Shahriari-Khalaji, Hamideh Valizadeh, Fariborz Sharifianjazi, Morteza Mehrjoo, Ketevan Tavamaishvili, Mohamadreza Tavakoli


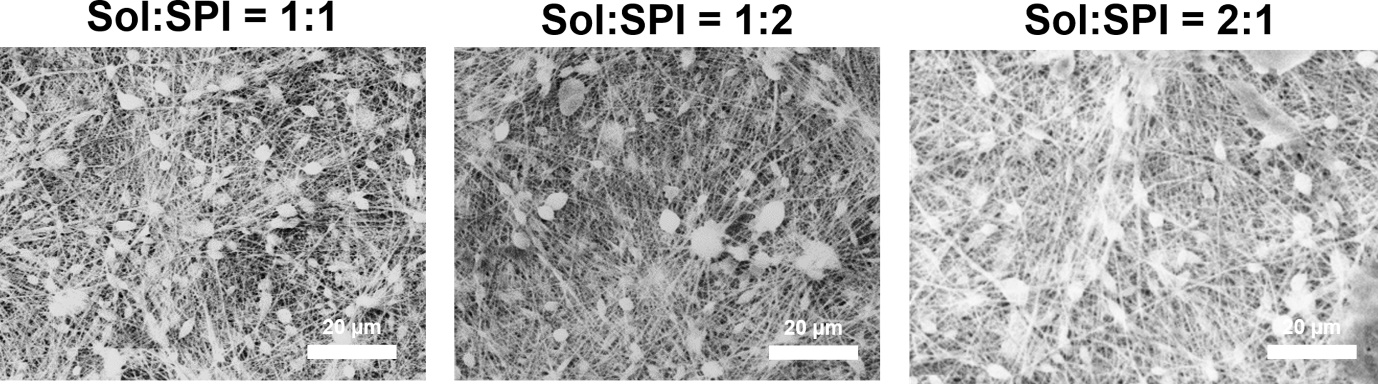


**Figure S1. SEM images of Sol/SPI nanofibers with different ratios**


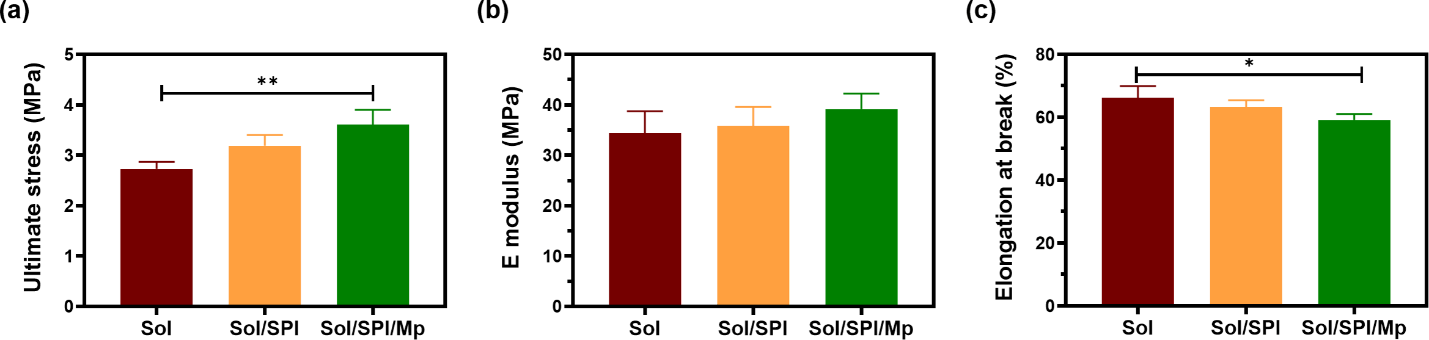


**Figure S2. Bar graphs related to mechanical properties of nanofibers**

**Table S1- Score of erythema and edema after application of the nanofibers.**

| Animal group | Skin reaction | Score after 24 h | Score after 48 h | Score after 72 h |
| --- | --- | --- | --- | --- |
| Sol | Erythema | 0 | 0 | 0 |
|  | Edema | 0 | 0 | 0 |
| Sol/SPI | Erythema | 0 | 0 | 0 |
|  | Edema | 0 | 0 | 0 |
| Sol/SPI/Mp | Erythema | 0 | 0 | 0 |
|  | Edema | 0 | 0 | 0 |
